# LC-MS Evaluation of the Redox Trypanothione Balance in *Leishmania infantum* Parasites

**DOI:** 10.3390/antiox14080977

**Published:** 2025-08-08

**Authors:** Théo Villarubias, Jade Royo, Pierre Perio, Sandra Bourgeade-Delmas, Jan Sudor, Alexis Valentin, Anne-Dominique Terrisse, Karine Reybier

**Affiliations:** Pharma-Dev UMR 152, Université de Toulouse, IRD, 118 Route de Narbonne, 31062 Toulouse CEDEX 9, France; theo.villarubias@univ-tlse3.fr (T.V.); jade.royo@univ-tlse3.fr (J.R.); pierre.perio@univ-tlse3.fr (P.P.); sandra.bourgeade-delmas@ird.fr (S.B.-D.); jan.sudor1@univ-tlse3.fr (J.S.); alexis.valentin@univ-tlse3.fr (A.V.); anne-dominique.terrisse@univ-tlse3.fr (A.-D.T.)

**Keywords:** *Leishmania infantum*, trypanothione redox balance, LC-MS analysis, oxidative stress, N-Ethylmaleimide

## Abstract

Leishmaniases are neglected tropical diseases caused by protozoan parasites of the *Leishmania* genus, with a significant global health burden, particularly in low-income regions. The parasites rely on a unique thiol-based redox system centered on trypanothione, which is essential for survival under oxidative stress encountered during their life cycle in both insect vectors and mammalian hosts. Given the absence of mammalian analogs, the trypanothione system represents an attractive target for antileishmanial drug development. However, accurate quantification of the reduced and oxidized forms of trypanothione has been challenging due to its instability and structural similarity between redox states. Here, we developed and validated a rapid, sensitive liquid chromatography–mass spectrometry (LC-MS) method for assessing the trypanothione redox state in *Leishmania infantum*. By incorporating N-ethylmaleimide as a thiol-blocking agent during sample preparation, the native redox state was preserved, enabling precise measurement of the reduced-to-oxidized ratio. Our approach demonstrated high sensitivity (nanomolar range), a rapid analysis time (5 min/sample), and robustness across various conditions. Moreover, we validated the method’s relevance in detecting oxidative stress and response to the trypanothione reductase inhibitor auranofin. This LC-MS technique provides a valuable tool for exploring *Leishmania* redox biology and supports the discovery of redox-targeting therapies against leishmaniasis.

## 1. Introduction

Leishmaniases are vector-borne diseases caused by parasites of the genus *Leishmania (Trypanosomatida: Trypanosomatidae)*, endemic to tropical and subtropical regions and the Mediterranean basin, and affecting 98 countries worldwide. These protozoan parasites are transmitted through the bite of infected female sandflies, which serve as the primary vectors for disease transmission in the Old and New World [1,2,3]. The World Health Organization (WHO) classifies leishmaniases as neglected tropical diseases, primarily affecting low-income populations globally. Current epidemiological data indicate that between 700,000 and 1 million new cases occur worldwide annually, resulting in approximately 20,000 to 30,000 deaths (Global Health Observatory data from WHO, 2018) [4]. Consequently, leishmaniases represent a significant public health challenge, with an increasing burden over the past decade. Among tropical infections, they rank as the second and fourth most common causes of death and disease, respectively [5].

Leishmaniases manifest in three primary clinical forms: cutaneous, mucocutaneous, and visceral, with the latter being the most severe and potentially fatal in 95% of cases if left untreated. Leishmaniases pose a significant health problem as the side effects associated with current therapies, as well as their high cost, limited efficacy, and route of administration, restrict their accessibility in developing countries. Additionally, the emergence of drug-resistant strains exacerbates this issue. Hence, there is an urgent need to find and design new antileishmanial drugs with improved safety profiles, efficacy, and accessibility. Different targets have been considered, such as specific enzymes or proteins that are essential for the parasite’s survival. Among druggable targets that have been more extensively studied are biomolecules involved in redox metabolism [6], which play crucial roles in parasite survival within hostile host environments. These include trypanothione synthetase, trypanothione reductase, and various peroxidases, all of which contribute to the unique redox system of trypanosomatid parasites.

*Leishmania* parasites undergo a complex digenetic life cycle involving two morphologically distinct forms. In the vector’s digestive tract, the parasites multiply as mobile, flagellated forms known as promastigotes, evolving from procyclic promastigotes in the midgut to metacyclic promastigotes in the insect’s anterior gut [7,8]. Upon inoculation into the mammalian host during the sandfly’s bloodmeal, promastigotes are rapidly internalized by macrophages, where they differentiate into a non-mobile form with a shortened flagellum, referred to as the amastigote form. During their life cycle, switching between mammals and insect vectors, *Leishmania* parasites must resist various oxidative stresses originating from the host’s immune response [9], especially from macrophages that generate superoxide anions (O_2_^•−^), hydrogen peroxide (H_2_O_2_), hydroxyl radicals (^•^OH), nitric oxide (NO^•^), and peroxynitrite (ONOO^−^). Furthermore, the parasite must adapt to modifications in temperature [10], pH and alterations in nutrient availability [11].

The mechanisms behind the parasite’s survival and evasion from these oxidative conditions are still not fully understood, but studies have demonstrated that *Leishmania* has developed an ingenious way to resist and maintain redox balance [11]. *Leishmania* and other trypanosomatids, unlike mammals, lack catalase, thioredoxin reductase, and glutathione peroxidase and reductase [12,13,14], which are key enzymes involved in detoxifying reactive oxygen species (ROS) in most eukaryotes. Instead, these parasites possess a unique redox-controlling system based on trypanothione (N1,N8-bis (glutathionyl) spermidine, T(SH)_2_) [15,16], a low-molecular-weight dithiol that functions as the central antioxidant in these organisms. This unique thiol system in trypanosomatids is maintained by a network of enzymes that include trypanothione synthetase, trypanothione reductase, tryparedoxin, and tryparedoxin peroxidase [17,18].

**Figure 1:**
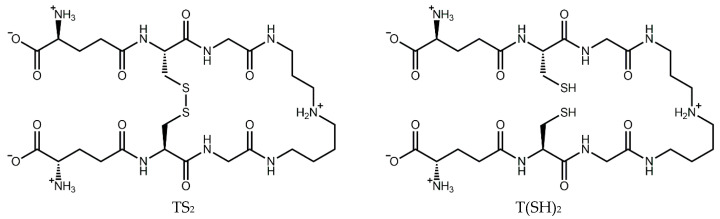
Structure of trypanothione in its oxidized (TS_2_) and reduced (T(SH)_2_) forms.

The trypanothione system operates in a cascade to ensure the homeostasis of this critical antioxidant. Trypanothione is synthesized by trypanothione synthetase from two molecules of glutathione (GSH) and one of spermidine. In the same manner as GSH, T(SH)_2_ can be oxidized by one- or two-electron oxidation mechanisms, ultimately leading to the formation of stable oxidized trypanothione (TS_2_) [16] (Figure 1). The reduced form can then be regenerated by trypanothione reductase in an NADPH-dependent reaction [19], completing the redox cycle. Ascorbate peroxidase and tryparedoxin peroxidase detoxify peroxides by oxidizing ascorbate and tryparedoxin, respectively. T(SH)_2_ then reduces dehydroascorbate and tryparedoxin, regenerating the antioxidant system [20].

The importance of this trypanothione-based system is underscored by genetic studies showing that disruption of genes encoding trypanothione synthetase or trypanothione reductase is lethal to the parasite, confirming the essential nature of this pathway [21,22]. Furthermore, studies have demonstrated that *Leishmania* parasites upregulate the expression of trypanothione synthesis and reduction enzymes during differentiation and in response to oxidative stress, highlighting the adaptive capacity of this system [23]. In the same way, *Leishmania* strains resistant to antimonial drugs exhibit increased expression of genes involved in thiol biosynthesis, including those for trypanothione synthesis and reduction [24].

This trypanothione-based system is at the core of the antioxidant defenses of the parasite. Therefore, a deeper understanding of the mechanisms that enhance its antioxidant system during infection is essential for developing new and effective treatments against *Leishmania*. The quantification of both reduced and oxidized forms of trypanothione represents a valuable approach to assess the redox status of the parasite under various conditions, including exposure to potential therapeutic agents.

Despite the biological and pharmacological significance of trypanothione, accurate measurement of its redox state in biological samples has been challenging due to the rapid oxidation of the reduced form during sample preparation, and the structural similarity between the reduced and oxidized forms. In this study, we therefore developed a liquid chromatography–mass spectrometry method for a rapid evaluation of the T(SH)_2_/TS_2_ balance, adapting a method from Moore et al. for GSH [25]. Our approach incorporates the use of *N*-Ethylmaleimide (NEM) as a thiol-blocking agent to prevent artifactual oxidation of T(SH)_2_ during sample processing, allowing for accurate determination of the native redox state. In addition, we validated the relevance of the T(SH)_2_/TS_2_ ratio as a marker of oxidative damage in *L. infantum* parasites subjected to oxidative stress and treatment with the trypanothione reductase inhibitor auranofin. This approach provides a valuable tool for studying the redox biology of *Leishmania* and evaluating potential therapeutic agents targeting the trypanothione system.

## 2. Materials and Methods

### 2.1. Parasites

*Leishmania infantum* (MHOM/MA/67/ITMAP-263) modified to express the luciferase gene was used for all experiments. This strain was originally isolated from a human case in Morocco in 1967 and has been extensively characterized in laboratory settings. The luciferase reporter gene integration allows for monitoring parasite viability through bioluminescence measurement, although this feature was not utilized in the current study. Procyclic promastigotes were cultured in RPMI 1640 with 25 mM HEPES (Biowest, Nuaillé, France) supplemented with 10% heat-inactivated fetal bovine serum (FBS, Biowest), 100 U/mL penicillin, 100 µg/mL streptomycin, 2 mM L-glutamine (VWR, Rosny-sous-Bois, France), and 50 µg/mL geneticin (Fisher Scientific, Pittsburgh, PA, USA) as a selection agent for the luciferase-expressing parasites, at pH 7.2, 24 °C. Cultures were maintained in logarithmic growth phase at 24 °C by biweekly dilution in fresh medium (2 × 10^6^ cells mL^−1^, final dilution). Cell density and viability were routinely monitored using a Neubauer hemocytometer and trypan blue exclusion.

### 2.2. Treatments

Promastigotes in their logarithmic growth phase (3–5 days post-inoculation, approximately 1–2 × 10^7^ cells·mL^−1^) were harvested via centrifugation at 3000× *g* for 10 min at room temperature, and resuspended in fresh RPMI 1640 medium without FBS at a density of 5 × 10^7^ cells·mL^−1^. The cell suspension was then distributed in 1 mL aliquots in sterile microcentrifuge tubes for subsequent treatments.

For oxidative stress experiments, parasites were exposed to hydrogen peroxide (Sigma-Aldrich, St. Louis, MO, USA) at a final concentration of 1 mM, or an equivalent volume of sterile water (H_2_O) as control, for 30 min, 1 h, or 2 h at 24 °C. The concentration of H_2_O_2_ was selected based on preliminary experiments that showed significant but non-lethal oxidative stress at this dose and time. For experiments involving auranofin, a gold-containing compound with known inhibitory activity against trypanothione reductase, parasites were pre-treated with 10 µM auranofin (Gold^I^(2S,3R,4S,5R,6R)-3,4,5-triacetyloxy-6-(acetyloxymethyl)oxane-2-thiolate tri-ethylphosphane), Sigma-Aldrich) dissolved in dimethyl sulfoxide (DMSO, Sigma-Aldrich), or DMSO alone as vehicle control, for 2 h at 24 °C, followed three washes in fresh culture medium. The samples were incubated in culture medium free of auranofin for 2 h, before being exposed to 1 mM H_2_O_2_ for 2 h. The final DMSO concentration in all cultures was maintained below 0.1% to avoid solvent-induced toxicity. Each experiment was carried out 3 to 4 times independently with triplicates for each condition.

At the designated time points, treated parasites were rapidly harvested by centrifugation at 3000× *g* for 5 min and washed once with 1 mL of ice-cold PBS to remove any residual treatment agents. The cellular pellet was then immediately subjected to the extraction procedure to minimize any artifactual changes in trypanothione redox state during sample processing.

### 2.3. Sample Preparation

For metabolite extraction, cell pellets were resuspended in 150 µL of pre-cooled (−20 °C) extraction solution under constant mixing using a vortex mixer. The basic extraction solution consisted of acetonitrile/methanol/water/DMSO (4:4:2:5, *v*/*v*/*v*/*v*) to facilitate cell lysis and protein precipitation. For samples intended for reduced trypanothione measurement, the extraction solution was supplemented with 1.67 mM *N*-Ethylmaleimide (Fisher Scientific) in DMSO as a thiol-blocking agent. NEM reacts specifically with free thiol groups to form stable thioether bonds, effectively preventing the oxidation of reduced trypanothione during sample processing.

Following initial resuspension, samples were vortexed vigorously for 10 s to ensure complete cell lysis. The samples were then left for one hour at −20 °C to allow for complete extraction while minimizing metabolite degradation or artifactual oxidation. After extraction, the samples were stored at −70 °C until analysis to ensure long-term stability of the extracted metabolites.

On the day of analysis, samples were thawed on ice and centrifuged at 20,000× *g* at 4 °C for 10 min to remove cellular debris and precipitated proteins. A volume of 100 µL of the clear supernatant was carefully collected, avoiding any pelleted material, and transferred into glass LC-MS vials with inserts suitable for low-volume samples. Vials were immediately placed in the refrigerated autosampler (4 °C) of the LC-MS system to prevent any degradation prior to analysis.

### 2.4. LC-MS Measurements

Liquid chromatography-mass spectrometry (LC-MS) analyses were performed using a comprehensive analytical system consisting of an Ultimate 3000 UHPLC system (Thermo Fisher Scientific, Courtaboeuf, France) coupled to a high-resolution LTQ-Orbitrap XL ETD mass spectrometer (Thermo Fisher Scientific, Courtaboeuf, France). The UPLC system included a solvent organizer SRD-3600 with integrated vacuum degasser for efficient removal of dissolved gases from mobile phases, a high-pressure binary gradient pump HPG-3400RS capable of delivering stable flows at the micro and analytical scale with minimal delay volume, a thermostated autosampler WPS3000TRS with precise injection control and sample temperature maintenance at 4 °C to prevent sample degradation, a column compartment oven TCC3000SD providing stable temperature conditions for chromatographic separations, and a UV–Visible detector DAD3000 for complementary spectrophotometric detection.

The mobile phases used for all analyses consisted of (A) 0.1% (*v*/*v*) formic acid prepared in ultrapure water (18.2 MΩ·cm, purified using a Milli-Q system) and (B) 0.1% (*v*/*v*) formic acid prepared in LC-MS grade methanol (Fisher Scientific). Both mobile phases were freshly prepared before each analysis batch and filtered through 0.22 µm membrane filters to remove any particulate matter that could interfere with the chromatographic separation or damage the analytical system.

Ultra-high-performance liquid chromatography (UPLC) was performed at a controlled temperature of 40 °C using a Luna Omega Polar C18 1.6 µm column (2.1 mm × 100 mm, Phenomenex, Torrance, CA, USA), which was selected for its excellent retention and separation of polar compounds such as trypanothione. A small injection volume of 2 µL was used to minimize band broadening while providing sufficient sensitivity. The flow rate was maintained at 200 µL/min using the following optimized chromatographic gradient conditions: initial composition of 95% A/5% B, which was maintained for 0.5 min to allow for adequate retention of polar compounds, followed by a linear increase to 95% B over 2.5 min, hold at 95% B for 1 min to ensure elution of any strongly retained compounds, then return to initial conditions (95% A/5% B) in 0.1 min, and finally re-equilibration at initial conditions for 0.9 min to ensure column stability for subsequent injections. The total run time was 5 min per sample.

Mass spectrometric detection was performed using heated positive electrospray ionization mode with the following source parameters optimized for maximum sensitivity: source voltage 5 kV, source vaporizer temperature 100 °C for thermal stability of the analytes, and a resolution setting of 7500 which provided adequate mass accuracy for the target compounds. The nebulization gas used for the analysis was nitrogen (N_2_). The sheath gas and auxiliary gas flow rates were set to 35 and 5 arbitrary units, respectively. The capillary temperature was set to 300 °C and capillary voltage to 35 V. The analysis was divided into two-time segments: 0–2.5 min and 2.5–5 min, with different scan events programmed for each segment to maximize sensitivity and specificity. For the first segment (0–2.5 min), two selective ion monitoring (SIM) events were programmed: SIM at *m*/*z* 722.55 ± 1.00 for the detection of TS_2_ and SIM at *m*/*z* 724.30 ± 0.50 for T(SH)_2_. For the second segment (2.5–5 min), a single SIM event at *m*/*z* 974.40 ± 0.50 was programmed for the detection of T(SNEM)_2_. This segmented approach allowed for optimal detection of both the reduced (NEM-derivatized) and oxidized forms of trypanothione with minimal interference.

All chromatographic and mass spectrometric data acquisition were performed using the Xcalibur software version 2.1 (Thermo Fisher Scientific), and data processing (including area under the curve (AUC) measurement) were performed using the Xcalibur software version 3.0 (Thermo Fisher Scientific). Analyte quantification was based on peak area measurements of the extracted ion chromatograms for the respective SIM channels, with external calibration using oxidized trypanothione standards.

### 2.5. Quantification Method

Before each measurement batch, an eight-point calibration curve of oxidized trypanothione (TS_2_) was generated over a concentration range of 10 nM to 5 µM, covering the expected physiological range in parasite samples. The oxidized trypanothione (TS_2_) standard solutions were freshly prepared by serial dilution of the stock solution (1.385 mM) in the extraction solvent acetonitrile/methanol/H_2_O/DMSO (4:4:2:5, *v*/*v*/*v*/*v*).

**Figure 2:**
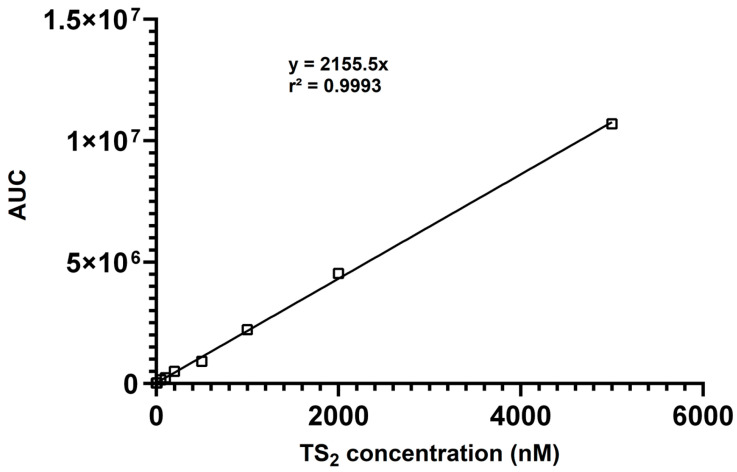
Calibration curve showing the linear responses of the AUC (area under curve) for TS_2_ (10–5000 nM).

The calibration data yielded excellent linearity (R^2^ = 0.9993) over the entire concentration range (Figure 2), demonstrating the suitability of the method for quantitative analysis. Linearity was confirmed by analyzing calibration standards in triplicate each day. The slopes and intercepts of the calibration curves were consistent, demonstrating the daily reproducibility of the method. The lower limits of detection (LLOD) and quantitation (LLOQ) were assessed by determining the lowest concentrations of TS_2_ that resulted in a signal-to-noise ratio of ≥3 (LLOD) and ≥10 (LLOQ), respectively. The LLOD and LLOQ values were confirmed through triplicate measurements and shown to be reproducible across three independent experiments. For TS_2_, the lower limit of detection (LLOD) was determined to be 3 nM, and the lower limit of quantitation (LLOQ) was 9 nM, indicating excellent sensitivity suitable for biological sample analysis.

Accuracy was evaluated at three concentration levels (30, 400, and 4000 nM) within the calibration range (10 nM–5 µM), and expressed as % bias. Precision, expressed as % RSD, was determined by repeatedly analyzing the full calibration range. Intra-day results showed an accuracy within ±8%, with precision (% RSD) below 20% at the limit of quantitation (LOQ).

The results are presented as a ratio relative to TS_2_ measured in the absence of NEM, which represents the total amount of trypanothione (oxidized and reduced) in the sample. This approach allows for direct comparison between samples, avoids the need for an internal standard, and ensures that matrix effects are automatically corrected.

### 2.6. Statistical Analyses

All data are presented as ratios of the chemical species concentrations to the concentration of total trypanothione. The data are expressed as the mean ± standard error of the mean (SEM) and were analyzed using GraphPad Prism software (version 10.4.2). *N* is the number of independent experiments performed and *n* is the number of replicates performed across all independent experiments. For the analyses of the concentrations of TS_2_ and T(SNEM)_2_ between parasites exposed to H_2_O_2_ and parasites exposed to ultrapure water overtime, a two-way ANOVA was performed before multiple comparisons by Tukey test between cells within the same row and the same column, where each row represents a different time point. For the analyses of the concentrations of TS_2_ and T(SNEM)_2_ in the auranofin experiment, a one-way ANOVA was performed before multiple comparisons via Tukey test between cells within the same row and the same column. Significant differences were defined as * *p* < 0.05, ** *p* < 0.01, *** *p* < 0.001, **** *p* < 0.0001.

## 3. Results

### 3.1. Method Development for Simultaneous Quantification of Reduced and Oxidiezd Trypanothione

The primary challenge in developing a reliable method for trypanothione redox state analysis was the inherent instability of the reduced form, which rapidly oxidizes to TS_2_ under ambient conditions, particularly at neutral pH and in the presence of oxygen. Preliminary experiments with standard extraction procedures revealed that only oxidized trypanothione could be detected in cell extracts, regardless of the actual redox state within the live parasites, indicating complete oxidation during the extraction process.

To address this challenge, we explored various thiol-blocking agents that could react rapidly with the free thiol groups of T(SH)_2_ to form stable derivatives before oxidation could occur. N-Ethylmaleimide was selected as the most suitable thiol-blocking agent based on its rapid reaction kinetics, high specificity for thiol groups, and stability of the resulting adducts. This blocker has been described for the measurement GSH [26,27]. NEM reacts with thiol groups via a Michael addition reaction to form stable thioether bonds, effectively preventing oxidation and allowing for the detection of the original reduced form as the NEM adduct (T(SNEM)_2_). The efficiency and completeness of the derivatization step were confirmed by LC-MS analysis. T(SNEM)_2_ was synthesized from T(SH)_2_ using (Tris(2-carboxyethyl)phosphine) (TCEP) as a reducing agent, followed by NEM-mediated derivatization. The reaction was shown to be rapid, completing within a few minutes, and the derivatized product (T(SNEM)_2_) remained stable for at least 48 h under the assay conditions.

The extraction solvent composition was optimized through a systematic evaluation of different solvent mixtures, considering factors such as extraction efficiency, protein precipitation, and compatibility with LC-MS analysis. A quaternary mixture of acetonitrile, methanol, water, and DMSO (4:4:2:5, *v*/*v*/*v*/*v*) was found to provide optimal extraction of trypanothione while effectively precipitating proteins and maintaining sample stability. The inclusion of DMSO, an uncommon component in typical LC-MS extraction solvents, was found to be critical for maximizing the extraction efficiency of trypanothione, possibly due to its ability to disrupt membrane structures and enhance solubilization of polar compounds. The extraction temperature was set at −20 °C to minimize metabolic activity during the extraction process, effectively “freezing” the metabolic state of the parasite at the time of harvest. The extraction procedure was further optimized by incorporating a brief sonication step, which improved extraction efficiency by enhancing cell lysis and release of intracellular metabolites.

**Figure 3:**
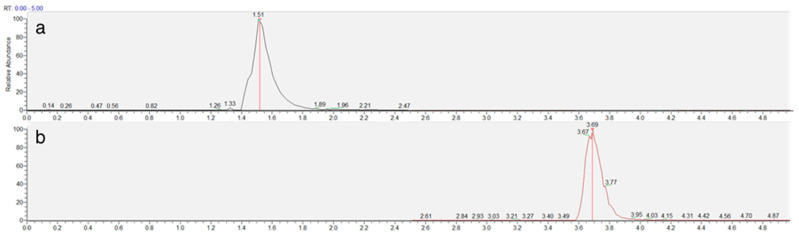
Representative extracted ion chromatogram of TS_2_ (**a**) and T(SNEM)_2_ (**b**) represented by time (min) and response (%) obtained from a cellular extract.

The chromatographic method was developed to achieve adequate separation of reduced (NEM-derivatized) and oxidized trypanothione within a reasonable analysis time. The Luna Omega Polar C18 column provided excellent retention and peak shape for both forms of trypanothione, with TS_2_ eluting at 1.5 min and T(SNEM)_2_ at 3.7 min under the optimized gradient conditions (Figure 3).

Quantification of the reduced form of trypanothione, present as the derivatized form (T(SNEM)_2_) after reaction with N-Ethylmaleimide, was carried out relative to the calibration curve of the oxidized form (TS_2_), the only commercially available form. Indeed, production of T(SNEM)_2_ standards from TS_2_ would have required elaborate sample preparation, including TCEP reduction, NEM derivatization, and removal of excess TCEP and NEM, as previously described for GSH [28].

### 3.2. Effect of NEM on Trypanothione Extraction and Measurement

**Figure 4:**
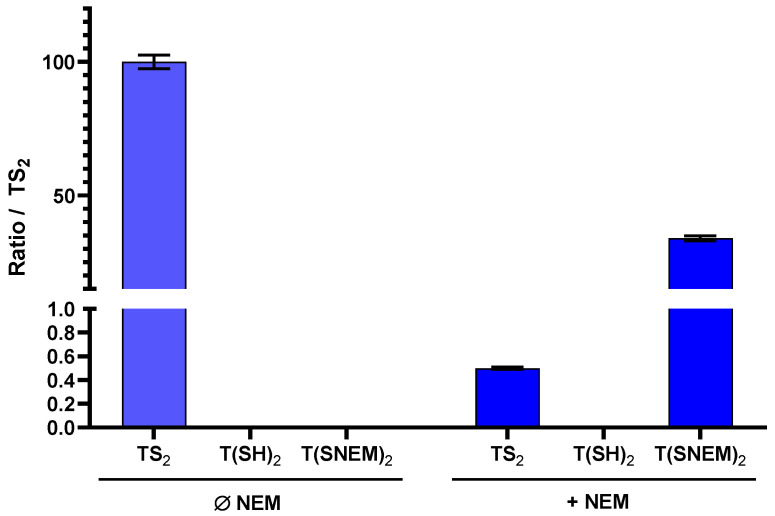
The effect of adding NEM to the extraction solution on the amount of oxidized TS_2_, reduced T(SH)_2_, and derivatized T(SNEM)_2_ after cellular extraction of *L. infantum* parasites. The results are presented as a ratio compared to TS_2_ measured in the absence of NEM representative of the total amount of trypanothione (oxidized or reduced) in the sample. Indeed, this amount varies from one sample to another, making the direct comparison of values not relevant (*N* = 3, *n* = 9).

To evaluate the effectiveness of NEM in preserving the redox state of trypanothione during sample preparation, we performed parallel extractions of *L. infantum* parasites with and without NEM in the extraction solvent. The results, presented in Figure 4, clearly demonstrate the critical importance of thiol blocking for the accurate assessment of trypanothione redox state.

In the absence of NEM, only TS_2_ was detected in the parasite extracts, with no detectable signal for T(SH)_2_. This observation is consistent with complete oxidation of the reduced form during the extraction process, which would lead to a significant overestimation of the oxidized form and prevent any assessment of the true intracellular redox state. In contrast, when NEM was included in the extraction solvent, both TS_2_ and the NEM-derivatized reduced form T(SNEM)_2_ were detected, with the latter predominating under normal physiological conditions. Notably, under basal conditions (untreated parasites), the amount of TS_2_ detected with NEM-containing extraction was very low (0.5%) compared to T(SNEM)_2_, indicating that the majority of trypanothione exists in the reduced state in healthy parasites. This finding is consistent with the established role of trypanothione as an active antioxidant, which requires the reduced form for its biological function.

A key methodological challenge encountered during method development was the discrepancy between the total trypanothione content (as measured by TS_2_ in the absence of NEM) and the sum of TS_2_ and T(SNEM)_2_ measured with NEM-containing extraction. Specifically, the sum of TS_2_ and T(SNEM)_2_ was consistently lower than the total TS_2_ measured without NEM, suggesting that the NEM-derivatized form was being underestimated when quantified against TS_2_ standards. This discrepancy was attributed to differences in ionization efficiency between TS_2_ and T(SNEM)_2_ in electrospray ionization mass spectrometry. The derivatized form, T(SNEM)_2_, exhibited significantly lower ionization efficiency compared to TS_2_, resulting in lower signal intensity for the same concentration. To account for this difference and enable accurate comparison of the two forms, we introduced a correction factor (k) calculated as follow:

k = ([TS_2_] − [TS_2_]_NEM_)/[T(SNEM)_2_], (1)

where [TS_2_] represents the total trypanothione measured without NEM, [TS_2_]_NEM_ represents the oxidized trypanothione measured with NEM, and [T(SNEM)_2_] represents the NEM-derivatized reduced trypanothione. To ensure reliability, the k value is determined from control experiments on the average of three independent experiments, each performed in triplicate (a total of 9 measurements).

### 3.3. Effect of H_2_O_2_ Treatment on Trypanothione Redox Status

To validate the biological relevance of our method and demonstrate its ability to detect changes in trypanothione redox state under conditions of oxidative stress, we exposed *L. infantum* promastigotes to hydrogen peroxide at a concentration of 1 mM for various durations (30 min, 1 h, and 2 h). H_2_O_2_ is a physiologically relevant oxidant that can directly oxidize thiol groups and is commonly used as a model compound for inducing oxidative stress in biological systems.

**Figure 5:**
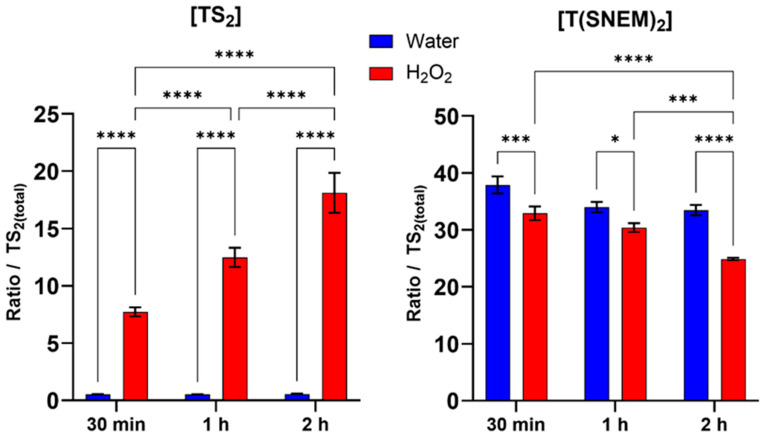
Impact of 30 min, 1 h, and 2 h H_2_O_2_ treatments on the amount of oxidized TS_2_ and reduced derivatized trypanothione T(SNEM)_2_. The results are presented as a ratio compared to TS_2_ measured in the absence of NEM. (*N* = 3, *n* = 9). Two-way ANOVA (Tukey test) * *p* < 0.05, *** *p* < 0.001, **** *p* < 0.0001.

The results, presented in Figure 5, showed a clear time-dependent shift in the trypanothione redox balance in response to H_2_O_2_ treatment. Compared to the water-treated control, H_2_O_2_-treated parasites exhibited a progressive increase in the level of oxidized trypanothione with increasing exposure time. This increase in TS_2_ was accompanied by a corresponding decrease in the level of reduced, NEM-derivatized trypanothione, consistent with the oxidation of reduced trypanothione by H_2_O_2_. Within the first 30 min of H_2_O_2_ exposure, a significant increase in TS_2_ levels and a corresponding decrease in T(SNEM)_2_ were observed compared to the water-treated control, indicating a rapid response to oxidative stress. This trend intensified with exposure time: after 1 h, TS_2_ levels continued to rise while T(SNEM)_2_ progressively declined. At 2 h of exposure, the redox imbalance was even more pronounced, with TS_2_ levels reaching nearly 20% of the total trypanothione pool, demonstrating substantial oxidation of the reduced form. In contrast, water-treated parasites maintained relatively stable levels of TS_2_ and T(SNEM)_2_ throughout the experiment, confirming that the observed changes were specifically due to the oxidative stress induced by H_2_O_2_.

For these experiments, the correction factor k was calculated from the values of [TS_2_] and [T(SNEM)_2_] of all control samples treated with ultrapure water. The resulting value was k = 2.857 ± 0.275. When expressed as the corrected ratio of reduced to oxidized trypanothione:

r = [T(SNEM)_2_] × k/[TS_2_] (2)

The data, calculated from three independent experiments, showed a decline from a mean value of approximately 174 in control conditions to a mean value of about 4 after 2 h of H_2_O_2_ exposure. This change in the redox ratio reflects the shift towards a more oxidized state under conditions of oxidative stress, demonstrating the sensitivity of the trypanothione system to oxidant challenge and the ability of our method to detect such changes.

The observed time-dependent oxidation of trypanothione in response to H_2_O_2_ is consistent with the established role of trypanothione as a primary antioxidant in *Leishmania* and particularly in the detoxification of H_2_O_2_. The reduced form (T(SH)_2_) reacts indirectly with H_2_O_2_ through peroxidases to neutralize the oxidant, resulting in the formation of TS_2_. Under normal conditions, TS_2_ would be rapidly reduced back to T(SH)_2_ by trypanothione reductase, maintaining the redox balance. However, under sustained oxidative stress, the rate of oxidation may exceed the reduction capacity, leading to the accumulation of the oxidized form and a shift in the redox ratio, as observed in our experiments.

### 3.4. Effect of Auranofin on Trypanothione Redox Balance During Oxidative Stress

To further validate our method and investigate the impact of disrupting the trypanothione redox cycle on the parasite’s response to oxidative stress, we evaluated the effect of auranofin, a gold-containing compound with known inhibitory activity against trypanothione reductase [29,30]. Trypanothione reductase catalyzes the NADPH-dependent reduction of TS_2_ to T(SH)_2_, a critical step in maintaining the reduced trypanothione pool necessary for antioxidant defense.

**Figure 6:**
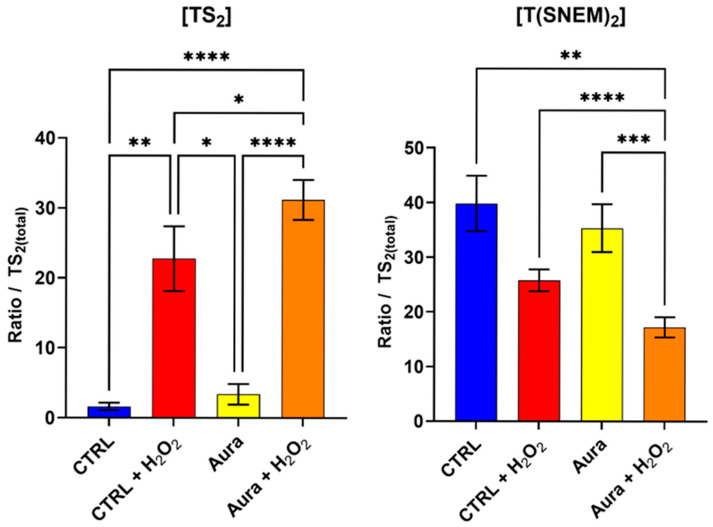
The effect of auranofin pre-treatment (2 h, 10 µM) on the amount of oxidized TS_2_ and reduced trypanothione T(SNEM)_2_. The results are presented as a ratio compared to TS_2_ measured in the absence of NEM. (*N* = 4, *n* = 12). One-way ANOVA (Tukey test) * *p* < 0.05, ** *p* < 0.01, *** *p* < 0.001, **** *p* < 0.0001.

Parasites were pre-treated with 10 µM of auranofin for 2 h to allow for sufficient inhibition of trypanothione reductase, followed by 2 h of exposure to 1 mM H_2_O_2_ to induce oxidative stress. This concentration of auranofin is similar to its IC_50_ (9.68 ± 1.02 µM) on *L. infantum* measured by Ilari et al. [30], thus ensuring an inhibitory effect. Control groups included parasites treated with auranofin alone, H_2_O_2_ alone, auranofin then H_2_O_2_, or neither (vehicle controls). The results, presented in Figure 6, reveal striking differences in the trypanothione redox response between auranofin-treated and untreated parasites when challenged with H_2_O_2_. In parasites pre-treated with auranofin followed by H_2_O_2_ exposure, the level of TS_2_was significantly higher compared to parasites treated with H_2_O_2_ alone. Conversely, the level of T(SNEM)_2_ was significantly lower in the auranofin + H_2_O_2_ group compared to H_2_O_2_ alone.

For these experiments, the correction factor k was calculated from the values of [TS_2_] and [T(SNEM)_2_] of all control samples treated with ultrapure water. The resulting value was k = 2.308 ± 0.595. When expressed as the corrected ratio of reduced to oxidized trypanothione, as in Equation 2, the data showed a decline from approximately 57 in control conditions to about 24 after 2 h of auranofin exposure. The ratio drops to 2.6 when control samples are exposed to 2 h of H_2_O_2_, and when cells treated with auranofin are exposed to H_2_O_2_, the ratio is even lower, at approximately 1.3. These enhanced oxidative shifts in the trypanothione redox balance in auranofin-treated parasites are consistent with the inhibition of trypanothione reductase, which would impair the parasite’s ability to regenerate the reduced form of trypanothione after oxidation by H_2_O_2_.

Interestingly, the parasites treated with auranofin alone (without H_2_O_2_ challenge) showed no significant changes in trypanothione redox state compared to the untreated controls. This observation suggests that under basal conditions, with minimal oxidative challenge, the partial inhibition of trypanothione reductase by the concentration of auranofin used (10 µM, 2 h) was not sufficient to disrupt trypanothione homeostasis. Yet, the ratio of reduced to oxidized forms of trypanothione was halved by this treatment, thus showing an effect nonetheless. The remaining enzyme activity, associated with a possible increase in trypanothione synthetase activity, was apparently sufficient to maintain the reduced trypanothione pool in the absence of additional oxidative stress [31].

## 4. Discussion

The method developed in this study represents a significant advancement in the quantitative analysis of trypanothione redox state in *Leishmania* parasites. By incorporating *N*-Ethylmaleimide as a thiol-blocking agent during the extraction process, we were able to preserve the native redox state of trypanothione, allowing for an accurate determination of both reduced and oxidized forms.

Our comparison of extractions with and without NEM (Figure 3) clearly demonstrated the critical importance of thiol blocking for accurate redox analysis. The complete oxidation of trypanothione observed in the absence of NEM highlights the extreme sensitivity of reduced trypanothione to oxidation during sample processing, emphasizing the need for immediate derivatization to preserve the native redox state. This finding has important implications for all studies involving thiol-based redox systems, not only in *Leishmania* but in any biological system where thiol oxidation during sample preparation can confound results.

The use of liquid chromatography coupled with high-resolution mass spectrometry provided excellent sensitivity and specificity for trypanothione analysis, with lower limits of detection and quantitation in the low nanomolar range. This sensitivity is particularly important for analyzing the trypanothione redox state in limited biological samples, such as clinical isolates or specific parasite stages that may be difficult to obtain in large quantities. Furthermore, the short analysis time (5 min per sample) allows for high-throughput screening, which is valuable for applications such as drug discovery where large numbers of compounds or conditions need to be evaluated.

A key methodological challenge addressed in our study was the difference in ionization efficiency between TS_2_ and T(SNEM)_2_ in mass spectrometry. The correction factor (k) we established provides a practical solution to this challenge, allowing for an accurate comparison of the two forms despite their different mass spectrometric responses.

While our method primarily focused on trypanothione as the major low-molecular-weight thiol in *Leishmania*, the same approach could potentially be extended to other thiols present in the parasite, such as glutathione, ovothiol, and various protein thiols. Such comprehensive thiol profiling would provide a more complete picture of the parasite’s redox network and how it responds to various stressors and interventions.

Furthermore, our approach offers advantages in terms of sample preparation simplicity, analysis time, and sensitivity. The single-step extraction procedure minimizes sample handling and the potential for artifactual oxidation, while the short LC-MS run time (5 min per sample) allows for high-throughput analysis. The nanomolar sensitivity achieved with our method permits analysis of small sample volumes, which is particularly valuable for limited biological specimens.

## 5. Conclusions

Our method for assessing the trypanothione redox state represents a significant advancement in the study of *Leishmania* redox biology. It provides a fast, simple, and reliable tool for basic research and for evaluating redox-targeting strategies in drug discovery focused on this unique parasite-specific pathway. The insights gained from this study contribute to the growing body of knowledge on trypanosomatid redox systems and support ongoing efforts to develop new therapies against these neglected parasitic diseases.

## Data Availability

Our dataset has been fully reviewed and published in the open data repository of the Université de Toulouse at the following DOI: https://doi.org/10.57745/HUZTJE.

## Abbreviations

The following abbreviations are used in this manuscript:

LC-MS	Liquid chromatography–mass spectrometry
NEM	N-ethylmaleimide
T(SH)_2_	Trypanothione
TS_2_	Trypanothione disulfide
T(SNEM)_2_	Trypanothione with NEM adduct
DMSO	Dimethyl sulfoxide
TCEP	Tris(2-carboxyethyl)phosphine
ROS	Reactive oxygen species
